# Supplementary material for: The histone variant H2A.W and linker histone H1 co-regulate heterochromatin accessibility and DNA methylation
Source: Nat Commun. 2021 May 11;12:2683. doi: 10.1038/s41467-021-22993-5 (PMC8113232; doi:10.1038/s41467-021-22993-5)
Supplement: Supplementary file 1 — Supplementary Information [file 41467_2021_22993_MOESM1_ESM.pdf]

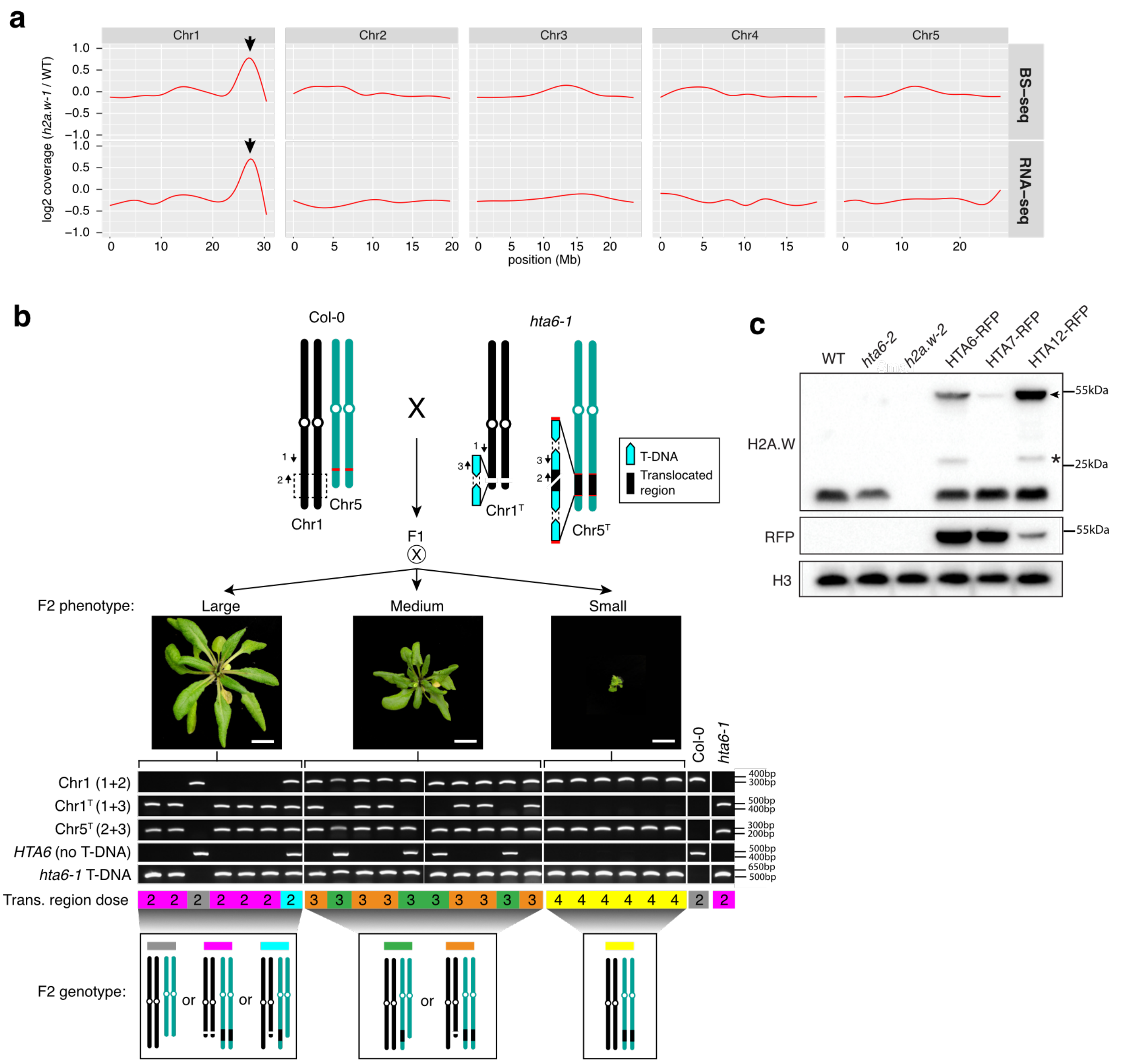

Supplementary Fig. 1. The *hta6-1* line contains a genomic rearrangement. a Sequencing coverage of published *h2a.w-1* BS-seq and RNA-seq data [1]. Locally weighted scatterplot smoothing (LOESS) fit of the log2 (*h2a.w-1* / WT) coverage ratio over non-overlapping 1 kb windows along the five *Arabidopsis* chromosomes. The black arrow indicates the genomic location of *CMT3*. **b** Scheme of a backcross between WT (Col-0) and *hta6-1.* Schematic representation of chromosomes 1 and 5 in the parental plants is shown. The *hta6-1* line harbors a large translocation of chromosome 1 to chromosome 5 linked to the *HTA6* gene (in red). A schematic representation of the T-DNA-associated rearrangements is shown. Partial characterization of these rearrangements was done by inverse PCR on *hta6-1*-circularized DNA after *Ssp*I digestion and by direct PCR amplification using primers specific to chromosome 1 and 5 and a T-DNA-specific primer (black arrows). On chromosome 5, the orientation of the chromosome 1 translation is unknown and is shown arbitrarily. The translocated part is flanked by T-DNA sequences at both sides in a likely complex genomic

structure as we were unable to PCR amplify chromosome 1 / chromosome 5 junctions. At chromosome 1, the ~5 Mb missing region is replaced by T-DNA/vector sequences. Plant pictures show representative images of the three plant phenotypes segregating in the F2 progeny of the Col-0 x *hta6-1* cross (scale bar = 1 cm). PCR analysis of the segregation of the *hta6-1* T-DNA and the chromosome 1 and 5 rearrangements in F2 plants of each phenotype is shown. Two independent experiments were performed with similar results. Genomic dose of the chromosome 1 translocated region is indicated below the gels, together with a schematic representation of the inferred corresponding chromosome 1 and 5 structures. **c** Western blot confirming the specificity of the H2A.W antibody. The antibody was tested on total nuclear extracts. No band is detected in *h2a.w-2* suggesting its specificity to H2A.W. Furthermore, the H2A.W antibody reacts to all H2A.W-RFP fusion proteins (bands indicated by the arrow) with different affinities, indicating that the antibody recognizes all three H2A.W variants. H3 antibody staining is carried out as loading control. Bands marked by an asterisk indicate a degradation product of H2A.W-RFP. Two independent experiments were performed with similar results. Source data for Supplementary Fig. 1a-c are provided as a Source Data file.

a

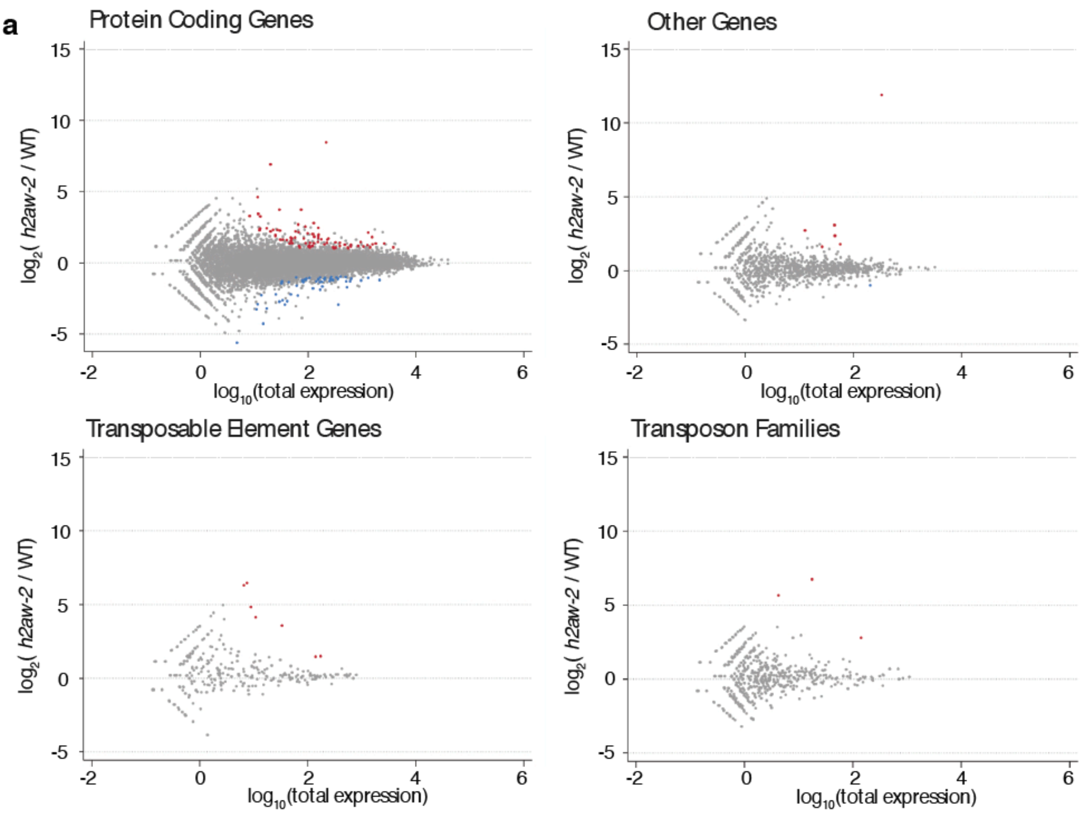

b

	h2a.w-2<WT	h2a.w-2>WT	not significant
PCG	52	78	23,374
TEG	0	7	1,315
other	1	6	2,037
TE	0	3	3,088

c

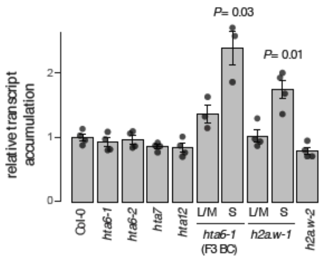

d

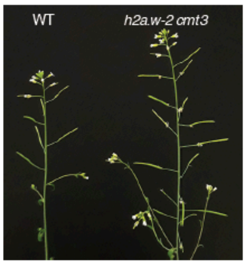

Supplementary Fig. 2. Transcriptional changes in *h2a.w-2*. a MA-plots of genes and TEs obtained using DEseq2 [2]. Genes were separated into protein coding, transposable element genes, and all other genes (ncRNAs, etc.). Significantly upregulated genes in *h2a.w-2* are highlighted in red, downregulated genes are in blue. **b** Summary of differentially expressed genes and TEs in *h2a.w-2*. **c** Quantification of *CMT3* transcripts by RT-qPCR in the indicated genotypes. L/M and S refer to Large/Medium and Small plant phenotypes, respectively (see Supplementary Fig. 1b). Plants with an S phenotype contain four doses of the chromosome 1 rearranged region. Statistically significant differences between the means from mutant and WT were tested with an unpaired two-sided Student's t-test. Statistically significant *p*-values (*: $P < 0.05$) are indicated. CMT3 transcript levels are normalized to *ACT2* and further normalized to Col-0. Sample means are shown with error bars representing standard error of the mean (n=3 biologically independent replicates for *hta6-1* F3 BC samples, n=4 biologically independent

replicates for others). **d** The *h2a.w-2 cmt3* quadruple mutants develop normally. Representative pictures of WT and *h2a.w-2 cmt3* plants.

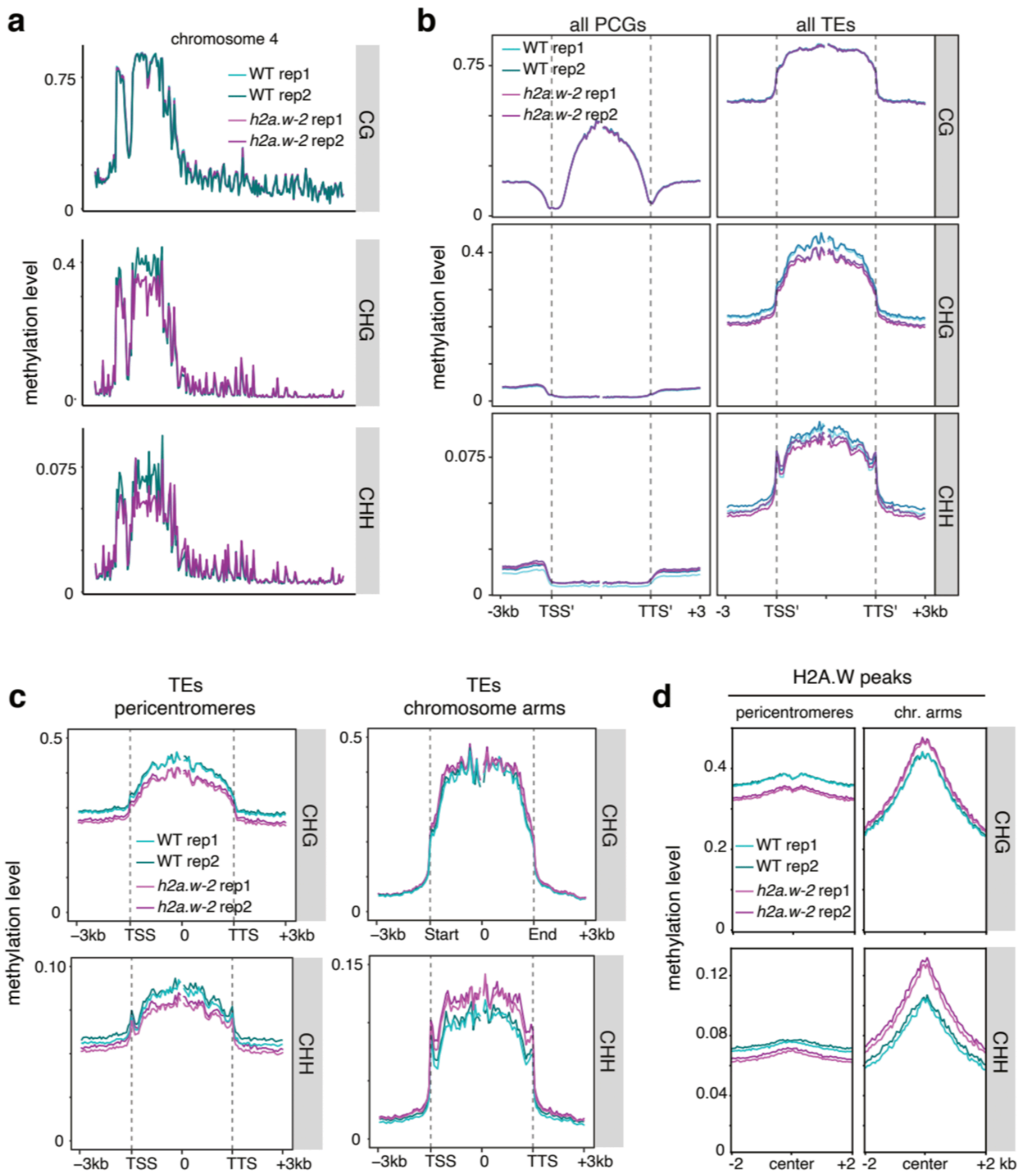

Supplementary Fig. 3. DNA methylation patterns in *h2a.w-2*. a Average DNA methylation levels in the CG, CHG, and CHH sequence contexts, in 100 kb windows across chromosome 4 in individual WT and *h2a.w-2* replicates. **b** CG, CHG, and CHH methylation levels over all Arabidopsis protein coding genes (PCGs) and all Arabidopsis TEs in WT and *h2a.w-2*. PCGs and TEs were aligned at the 5’ (left dashed line) and 3’ end (right dashed line), and sequences 3 kb upstream or downstream were included, respectively. Average methylation over 100 bp bins is plotted. **c** Metaplots of CHG and CHH methylation levels over TEs located in pericentromeric heterochromatin and TEs located in chromosome arms in WT and *h2a.w-2*. **d** CHG and CHH methylation levels over H2A.W peaks in the chromosome arms and pericentromeres.

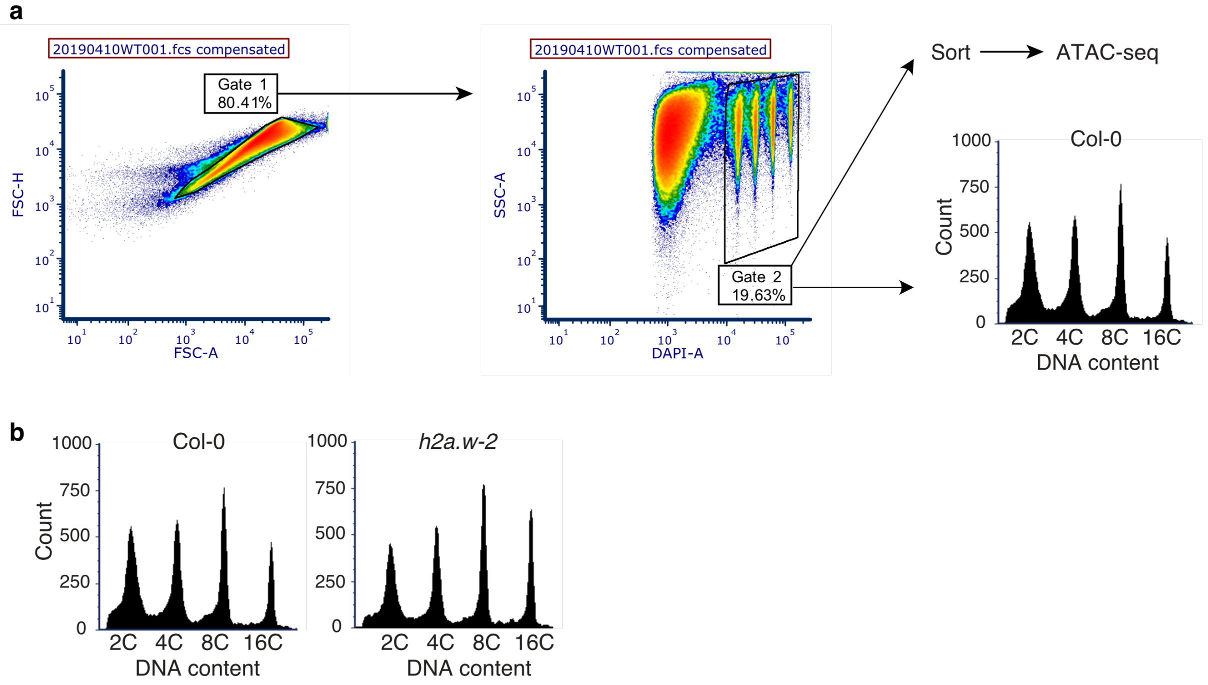

Supplementary Fig. 4. *h2a.w-2* does not cause endoreduplication defects. a FACS gating/sorting strategy scheme used for nuclei analysis and sorting. **b** Flow cytometry profiles of nuclei isolated from 10-day old seedlings of Col-0 and *h2a.w-2.* The X-axis represents ploidy level or DNA content and the Y-axis represents the number of nuclei.

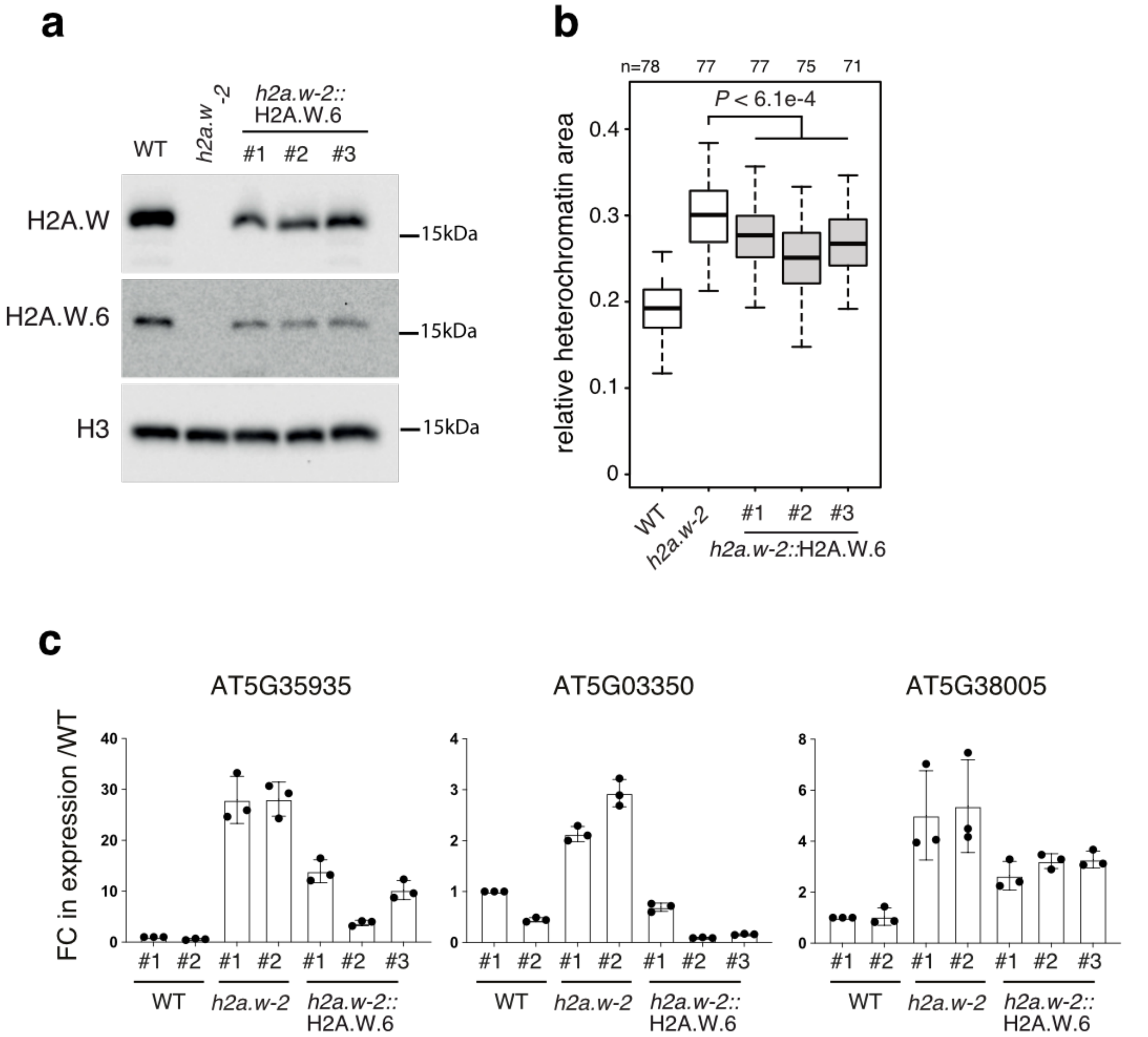

Supplementary Fig. 5. Complementation of *h2a.w-2*. a Western blot confirming expression of H2A.W.6 in three *h2a.w-2* complementation lines and WT and *h2a.w-2* controls. Two independent experiments were performed with identical results. **b** Partial rescue of relative chromocenter fraction in H2A.W.6-complemented *h2a.w-2* lines. Number of analyzed nuclei are indicated on the top. Whiskers indicate 1.5X IQR. Outliers are represented by circles. Relative chromocenter fraction in rescued lines significantly lower than in *h2a.w-2* (P = 6.1e-4, two-sided Wilcoxon rank sum test). **c** Relative expression of three genes upregulated in *h2a.w-2* in WT, *h2a.w-2*, and *h2a.w-2*::H2A.W.6 by qPCR. Source data for Supplementary Fig. 5a are provided as a Source Data file.

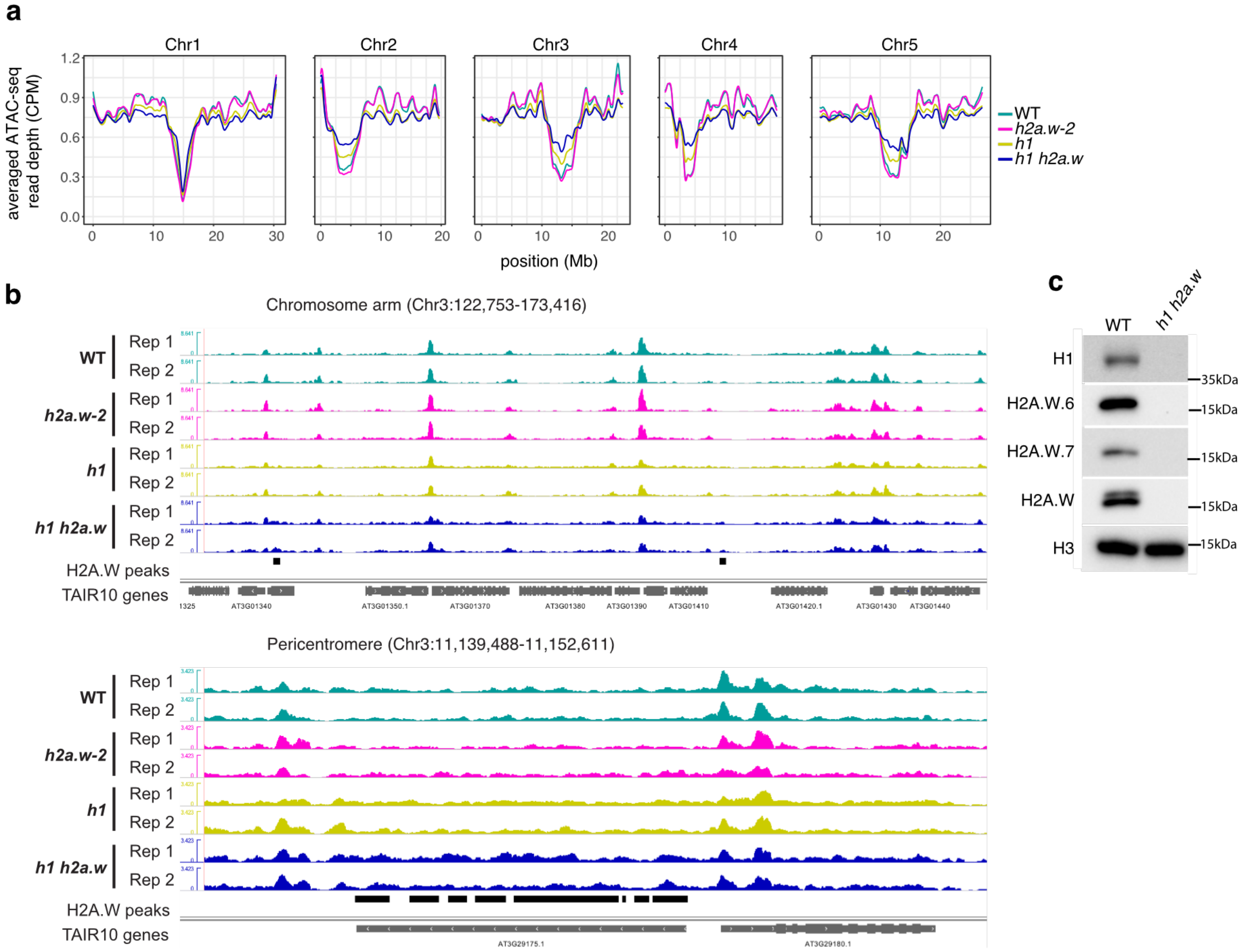

Supplementary Fig. 6. H1 and H2A.W regulate heterochromatin accessibility. **a** Locally weighted scatterplot smoothing (LOESS) fit of ATAC-seq read depth averaged in 50 kb bins across all *Arabidopsis* chromosomes in indicated genotypes. Average of two replicates is shown. **b** Genome browser views of genomic regions on the left arm (top) and on the pericentromere (bottom) of chromosome 3. The two ATAC-seq replicates for each genotype are shown. **c** Western blot confirming *h1 h2a.w* quintuple loss of function mutant. Two independent experiments were performed with identical results. Source data for Supplementary Fig. 6c are provided as a Source Data file.

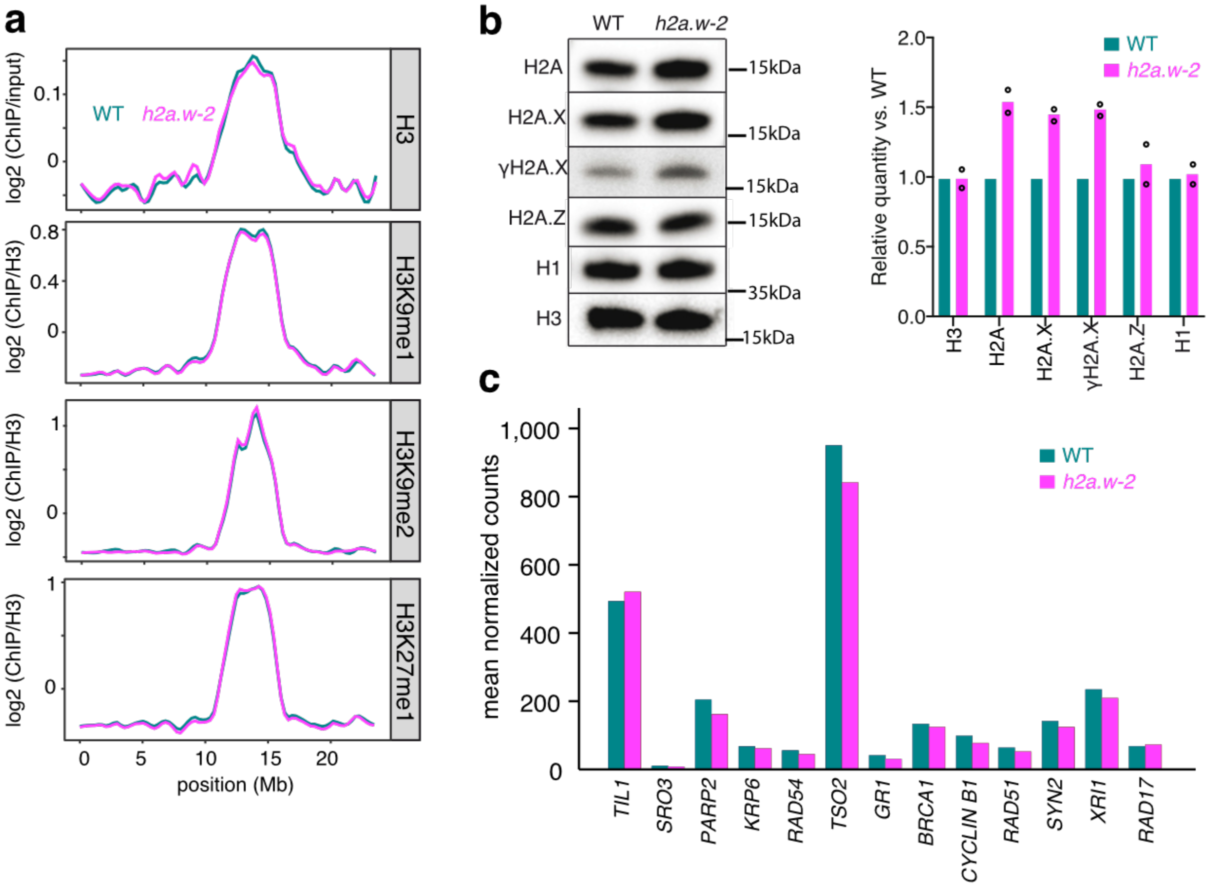

Supplementary Fig. 7. Patterns of heterochromatic H3 modifications and accumulation of H2A variants in *h2a.w-2*. a Locally weighted scatterplot smoothing (LOESS) fit of H3, H3K9me1, H3K9me2, and H3K27me1 levels averaged in 1 kb bins across chromosome 3 in WT and *h2a.w-2*. Average of two replicates shown. **b** Western blot comparing replicative H2A, H2A.X, γH2A.X, H2A.Z, and H1 protein levels between Col-0 and *h2a.w-2*. Western was performed on nuclear extracts. H3 is used as a loading control. Bar plot represents the quantification of protein levels. The plot was generated using data from two independent experiments. **c** Comparison of the expression levels of 13 genes upregulated in response to DNA damage [3] in *h2a.w-2* and WT. Source data for Supplementary Fig. 7b are provided as a Source Data file.

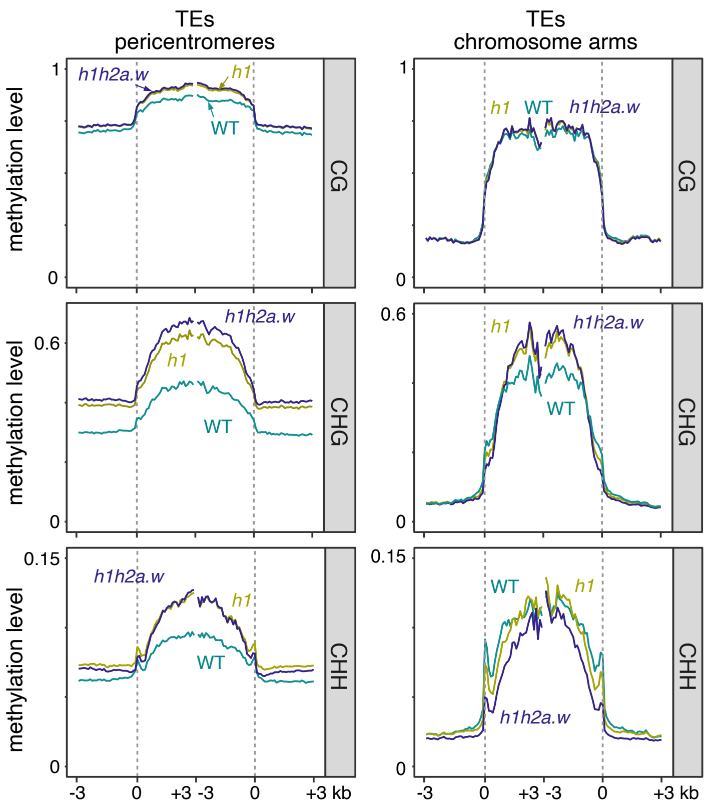

Supplementary Fig. 8. DNA methylation profiles at TEs in *h1* and *h1 h2a.w.* CG, CHG, and CHH methylation levels over TEs in pericentromeres and chromosome arms in WT, *h1* and *h1 h2a.w*. TEs were aligned at the 5' (left dashed line) and 3' end (right dashed line), and sequences 3 kb upstream or downstream were included, respectively. Average methylation over 100 bp bins is plotted.

```
H1.1    MSEVEIENAATIEGNTAADAPVTDAAVEKKPAAKG---RKTKNVKEVKEKKTVAAAPKKR 57
H1.2    -MSIEEENVPTTVDSGAADTTVK--SPEKKPAAKGGKSKKTTTAKATKKPVKAAAPTKKK 57
          .:* **..*  .. ***:.*.  : ********   :**...* .*:  ..**..**:

H1.1    TVSSHPTYEEMIKDAIVTLKERTGSSQYAIQKFIEEKRKELPPTFRKLLLLNLKRLVASG 117
H1.2    TTSSHPTYEEMIKDAIVTLKERTGSSQYAIQKFIEEKHKSLPPTFRKLLLVNLKRLVASE 117
        *.***********************************:*.**********:********

H1.1    KLVKVKASFKLPSASAKAS-SPKAAAEKSAPAKKKP-ATVAVTKAKRKVAAASKAKKTIA 175
H1.2    KLVKVKASFKIPSARSAATPKPAAPVKKKATVVAKPKGKVAAAVAPAKAKAAAKGTKKPA 177
        **********:*** : *: .* *..:*.*..  ** ..**.: *  *. **:*..*. *

H1.1    VKPKTAAAKKVTAKAK---AKPVPRATAAATKRKAVDAKPKAKARPAKAAKTAKVTSPAK 232
H1.2    AKVVAKAKVTAKPKAKVTAAKPKSKSVAAVSKTKAVAAKPKAKERPAKASRTSTRTSPGK 237
        .*  : *  ....***   *** .::.**.:* *** ****** *****::*:. ***.*

H1.1    KAVAATKKVATVATKKKTPVKKVVKPKTVKSPAKRASSRVKK- 274
H1.2    KVAAPAKKVAVT---KKAPAKSVK----VKSPAKRASTRKAKK 273
        *..*.:****..   **:*.*.*     *********:*  *
```

Supplementary Fig. 9. Alignment of *Arabidopsis* Histone H1.1 and H1.2. The alignment was generated using Clustal Omega (https://www.ebi.ac.uk/Tools/msa/clustalo/). SPKK-like motifs in C-terminal tails of H1 are highlighted in blue.

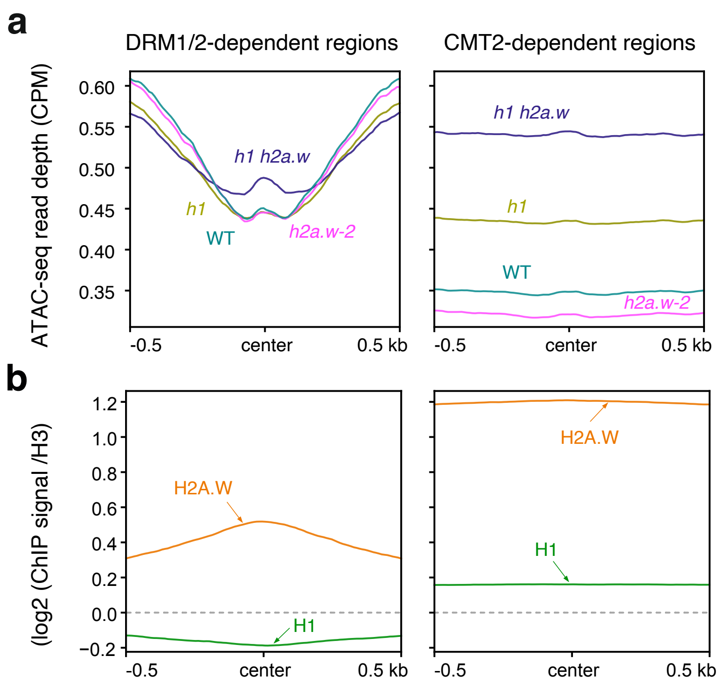

Supplementary Fig. 10. Chromatin accessibility and H2A.W and H1 enrichment at DMR1/2- and CMT2-dependent regions. a ATAC-seq read depth over DRM1/2- and CMT2-dependent regions in the indicated genotypes. Average of two replicates shown. **b** Wild-type levels of H2A.W and H1 over DRM1/2- and CMT2-dependent regions.

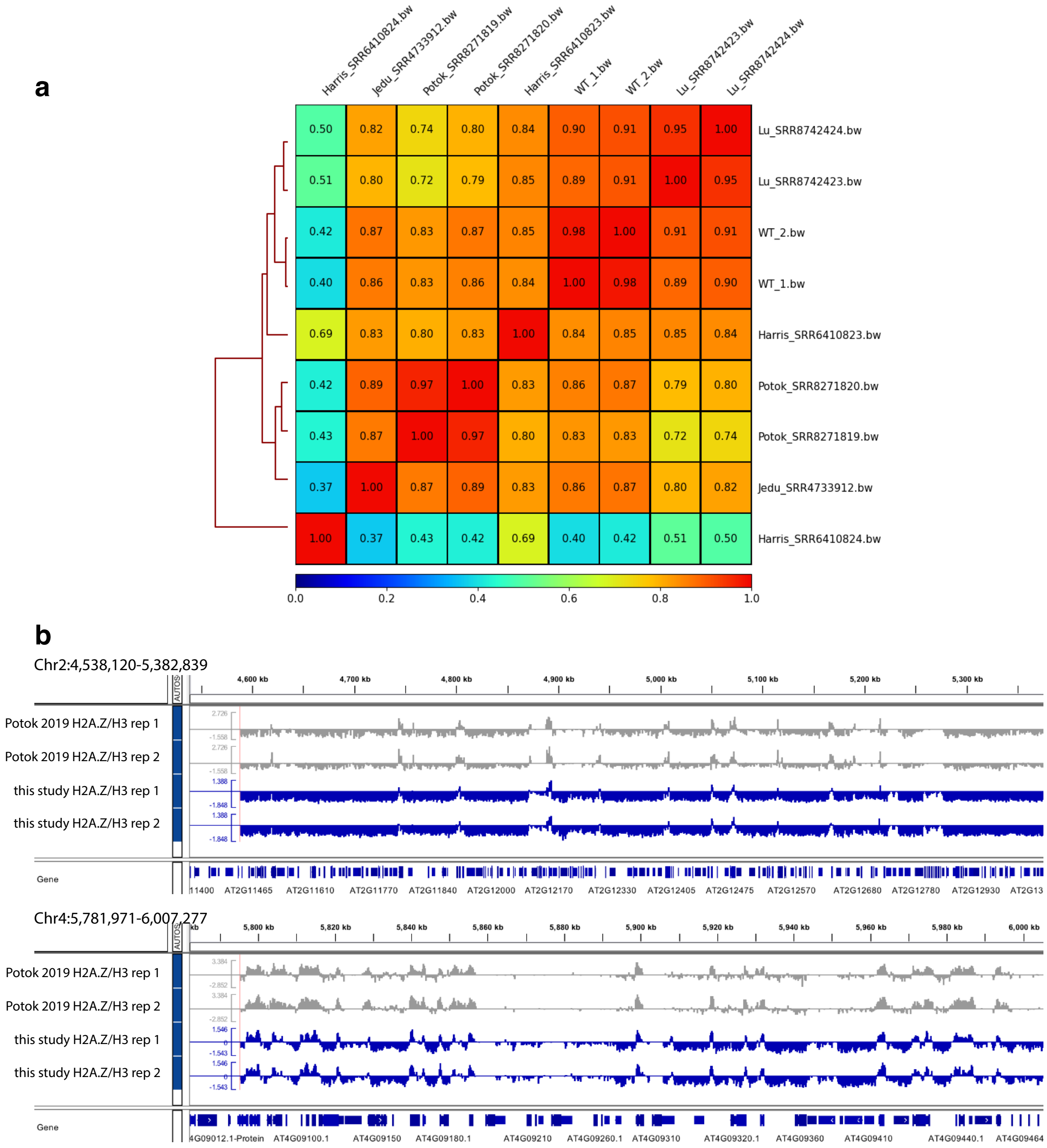

Supplementary Fig. 11. Comparison of ATAC-seq and H2A.Z ChIP-seq data generated in this study with previously published data. **a** Hierarchical heatmap of ATAC-seq replicates described in this study (WT_1 and WT_2) and published ATAC-seq datasets based on Spearman correlation coefficients. Clusters were constructed using complete linkage. Colors represent the correlation coefficients that are also indicated in each box. **b** Genome browser views of H2A.Z ChIP-seq replicates described in this study and H2A.Z ChIP-seq replicates published in Potok et al 2019 [4]

Supplementary Table 1. List of TEs with significantly increased copy number in *h2a.w-1* vs. WT BS-seq data [1]

Chromosome	Start	End	TE accession	Strand	WT (RPM)	*h2a.w-1* (RPM)	Log_2 ratio (*h2a.w-1* / WT)	*P* val
Chr1	24 604 957	24 605 560	AT1TE80825	+	6.72	20.48	1.61	0.0077
Chr1	24 704 986	24 706 028	AT1TE81150	+	7.47	22.04	1.56	0.0066
Chr1	25 159 042	25 163 500	AT1TE82600	+	59.64	145.89	1.29	0.0000
Chr1	25 180 003	25 182 025	AT1TE82640	+	9.28	25.23	1.44	0.0072
Chr1	25 639 634	25 641 849	AT1TE84070	-	10.22	27.76	1.44	0.0061
Chr1	26 030 838	26 036 307	AT1TE85290	-	43.49	88.85	1.03	0.0001
Chr1	26 366 602	26 371 673	AT1TE86360	+	60.23	154.77	1.36	0.0000
Chr1	26 374 207	26 375 957	AT1TE86375	+	10.53	28.95	1.46	0.0041
Chr1	26 779 007	26 781 652	AT1TE87630	-	11.56	28.01	1.28	0.0075

Supplementary Table 2. List of primers used in this study.

target	name	primer sequence	comment
**RTqPCR / qPCR**			
CMT3	CMT3 qPCR pri3F	GACGTGCGATTGTTGATGAG	
	CMT3 qPCR pri3R	AGTTTCCCATTAGCCCCTTC	
TSI	TSIqF	CTCTACCCTTTGCATTCATGAATCCTT	from Pecinka et. al. Plant Cell 2010
	TSIqR	GATGGGCAAAAGCCCTCGGTTTTAAAATG	
ACT2	act2pri3F	GGCTTAAAAAGCTGGGGTTT	
	act2pri3R	TTGTCACACACAAGTGCATCA	
MULE	MULEqF	GGCACTTCAATTGTGCTTTTTCCT	
	MULE R2	GATACTTGTTGACAAGTGTTTAGCAAGCC	
45S rDNA	18S qF	AAACGGCTACCACATCCAAG	
	18S qR	CCTCCAATGGATCCTCGTTA	
180bp	180all-F	ACCATCAAAGCCTTGAGAAGCA	
	180all-R	CCGTATGAGTCTTTGTCTTTGTATCTTCT	
**Genotyping**			
hta6-1	H2A.W.6-TDNA-RP	AATACATAGTCACGCGGATCG	
	hta6 screen R	GCTCCAGAACCTGAGATCAATAA	
	LBb1	GCGTGGACCGCTTGCTGCAACT	
hta6-2	1931	AATTTCAGTAATCGATAACCGTAGC	BsaBI cuts mutant amplicon
	1932	CTTCCCACTGGGAATTGAAGACCGGaT	
hta7	H2A.W.7_GABI_149G05_LP	TTAAATTCTAAACCCCACCGG	
	H2A.W.7_GABI_149G05_RP	TTTGGAGCTTTTGAACAATGG	
	GABI-Kat-LB	ATATTGACCATCATACTCATTGC	
hta12	H2A.W.12_SAIL_667_D09_LP	AGTTTCCTGTCGGTAGGATCG	
	H2A.W.12_SAIL_667_D09_RP	TCAGTTTTGTTTTTCCATCGG	
	LB3	TAGCATCTGAATTTCATAACCAATCTCGATACAC	
chr1 WT	(1) hta6 no rearran F1	ATCTGCAGTTTAATTTAGTTCACCA	
	(2) hta6R3	AGGATAACCAGTGAATCCATTTTGC	
chr1T	(1) hta6 no rearran F1	ATCTGCAGTTTAATTTAGTTCACCA	primers numbered as in Supplementary figure 1b
	(3) LBb1	GCGTGGACCGCTTGCTGCAACT	
chr5T	(2) hta6R3	AGGATAACCAGTGAATCCATTTTGC	
	(3) LBb1	GCGTGGACCGCTTGCTGCAACT	
**Translocation**			
southern blot probe	1815	CGTGCATATGACGAACACAC	
	1816	CACCGCAATAATTGATGCTC	
**Sequence characterization of rearrangement junctions**			
chr1T left border	hta6 no rearran F1	ATCTGCAGTTTAATTTAGTTCACCA	
	LBb1	AGGATAACCAGTGAATCCATTTTGC	
chr1T right border	hta6 transloc R1	CAACGACGAAGCTTTGGCTC	
	LBb1	GCGTGGACCGCTTGCTGCAACT	
chr5T left border	H2A.W.6-TDNA-LP	AATCGCAAAACATCGTAATGG	
	LBb1	GCGTGGACCGCTTGCTGCAACT	
chr1 tanslocation left border (iPCR)	hta6 F2	TGTTCTCATTTTGGCCAATGATTT	1st round amplification iPCR
	hta6 R2	ATCCAAGTACAGACAGCCATCG	
	hta6 F1	TGGCCAATGATTTATCGGCTAC	2nd round amplification iPCR
	hta6 R3	AGGATAACCAGTGAATCCATTTTGC	
chr1 translocation right border	hta6 transloc F1	GGCCGATTCCCACAAATTAGC	
	LBb1	GCGTGGACCGCTTGCTGCAACT	
**CRISPR/Cas9**			
CRISPR/Cas9 guide RNA	HTA6 gRNA1-2F	ATTGGTTTCGAAATCGATGAAAGC	Annealed oligonucleotides for *Bbs* I insertion in the pEN-Chimera vector
	HTA6 gRNA1-2R	AAACGCTTTCATCGATTTCGAAAC	
screening for *HTA6* mutations	1871	TCACAAATCTTCAACTTCCGATA	PCR amplification for sequencing
	1872	CGTATCGTCCTTTCTTCAGGA	

Supplementary Table 3. Total read counts and mapping statistics of RNA-seq data.

sample	PE reads sequenced	pass QC/filtering	align uniquely	align uniquely, PCR duplicates removed
WT rep #1	36 154 621	35 713 465	32 337 973	14 201 090
WT rep #2	28 381 071	27 895 725	25 525 526	9 881 399
WT rep #3	37 145 738	36 526 846	33 580 562	14 500 730
h2a.w-2 rep #1	31 790 016	31 287 974	28 555 127	13 051 555
h2a.w-2 rep #2	35 413 449	34 792 988	31 758 032	9 878 738
h2a.w-2 rep #3	33 221 095	32 794 067	29 983 389	13 162 519

Supplementary Table 4. Total read counts and mapping statistics of ATAC-seq data.

sample	PE reads sequenced	pass QC/filtering	align uniquely	align uniquely, PCR duplicates removed
WT rep #1	38 590 783	38 405 068	23 098 444	10 468 189
WT rep #2	55 673 712	55 401 220	32 329 479	14 349 096
h2a.w-2 rep #1	40 574 799	40 369 801	24 002 790	12 234 144
h2a.w-2 rep #2	40 122 757	39 946 855	24 044 620	11 671 421
h1 rep #1	73 135 956	72 401 577	40 674 759	16 717 663
h1 rep #2	38 030 217	37 846 268	22 949 557	12 364 826
h1 h2a.w-2 rep #1	39 586 172	39 384 818	23 393 315	11 883 424
h1 h2a.w-2 rep #2	36 944 619	36 770 899	21 885 131	11 020 020

Supplementary Table 5. Total read counts and mapping statistics of BS-seq data.

	PE reads sequenced	pass QC/filtering	align uniquely	align uniquely, PCR duplicates removed
WT rep #1	34 007 928	33 902 872	22 747 873	18 491 516
WT rep #2	32 165 662	32 043 241	20 362 930	16 065 050
h2a.w-2 rep #1	34 050 262	33 945 360	22 430 253	17 854 255
h2a.w-2 rep #2	32 093 502	31 969 711	19 938 220	15 950 267
WT rep #3	143 006 865	142 462 588	110 631 899	91 322 705
WT rep #4	139 932 996	139 501 891	95 334 514	76 310 941
h1 rep #1	127 354 430	126 863 199	78 756 889	63 005 125
h1 rep #2	126 574 188	126 093 206	78 773 478	65 815 115
h1 h2a.w rep #1	134 798 590	134 302 531	82 011 595	66 521 520
h1 h2a.w rep #2	123 315 213	122 836 750	77 835 020	63 942 635

Supplementary Table 6. Total read counts and mapping statistics of ChIP-seq data.

genotype	antibody	replicate	sample	PE reads sequenced	pass QC/filtering	align uniquely	align uniquely, PCR duplicates removed
WT	H1	1	WT H1 rep1	18059253	18019659	14036214	12743771
WT	H1	2	WT H1 rep2	35733897	35290905	27827834	24392726
WT	H2A	1	WT H2A rep1	31230251	31151691	26140649	20958323
WT	H2A	2	WT H2A rep2	17982412	17207724	14361165	12924478
WT	H2A.X	1	WT H2A.X rep1	15629566	15435287	13285545	11574043
WT	H2A.X	2	WT H2A.X rep2	12804074	11845502	10151612	9306136
WT	H2A.Z	1	WT H2A.Z rep1	21052748	21033103	19940916	17747106
WT	H2A.Z	2	WT H2A.Z rep2	25546975	24942044	23678196	20948729
WT	H3	1	WT H3 rep1	17766125	17752129	14274871	13090605
WT	H3	2	WT H3 rep2	32366387	31988521	26007638	23201032
WT	H3K27me1	1	WT H3K27me1 rep1	28612351	28210715	18271872	13577539
WT	H3K27me1	2	WT H3K27me1 rep2	19331994	16318507	10183947	8339032
WT	H3K9me1	1	WT H3K9me1 rep1	19931216	19889606	13588202	11749541
WT	H3K9me1	2	WT H3K9me1 rep2	28802444	28357626	19176612	16125231
WT	H3K9me2	1	WT H3K9me2 rep1	11452267	10703834	5976783	4102263
WT	H3K9me2	2	WT H3K9me2 rep2	8645986	4694581	2253810	1872079
WT	input	1	WT input rep1	26239514	26226328	21088112	18113794
WT	input	2	WT input rep2	26260379	25930142	21242190	18258221
h2a.w-2	H1	1	h2a.w-2 H1 rep1	14062552	14032895	10469994	9477896
h2a.w-2	H1	2	h2a.w-2 H1 rep2	27074404	26638350	20056301	17684994
h2a.w-2	H2A	1	h2a.w-2 H2A rep1	28296510	28244375	21245010	17614648
h2a.w-2	H2A	2	h2a.w-2 H2A rep2	19976756	18973675	14255164	12539325
h2a.w-2	H2A.X	1	h2a.w-2 H2A.X rep1	26799783	26679591	19136370	15846497
h2a.w-2	H2A.X	2	h2a.w-2 H2A.X rep2	16724550	15934522	11365928	9979999
h2a.w-2	H2A.Z	1	h2a.w-2 H2A.Z rep1	25002697	24967989	23299297	20468667
h2a.w-2	H2A.Z	2	h2a.w-2 H2A.Z rep2	22042538	21238200	19816893	17837550
h2a.w-2	H3	1	h2a.w-2 H3 rep1	15299993	15286081	11872925	10380528
h2a.w-2	H3	2	h2a.w-2 H3 rep2	27108223	26770710	21114372	18879384
h2a.w-2	H3K27me1	1	h2a.w-2 H3K27me1 rep1	26918379	26867093	16289178	11881435
h2a.w-2	H3K27me1	2	h2a.w-2 H3K27me1 rep2	21084530	17782880	10551938	8402490
h2a.w-2	H3K9me1	1	h2a.w-2 H3K9me1 rep1	12185166	12164444	8119754	7107167
h2a.w-2	H3K9me1	2	h2a.w-2 H3K9me1 rep2	19859654	19569388	12696388	10757102
h2a.w-2	H3K9me2	1	h2a.w-2 H3K9me2 rep1	12903492	12775666	6135482	4211550
h2a.w-2	H3K9me2	2	h2a.w-2 H3K9me2 rep2	12597300	7129708	2850112	2271449
h2a.w-2	input	1	h2a.w-2 input rep1	26683510	26649025	21203377	18196541
h2a.w-2	input	2	h2a.w-2 input rep2	14603694	14206285	11301655	10035176

Supplementary References

1. Yelagandula, R. *et al.* The histone variant H2A.W defines heterochromatin and promotes chromatin condensation in arabidopsis. *Cell* **158**, 98–109 (2014).
2. Love, M. I., Huber, W. & Anders, S. Moderated estimation of fold change and dispersion for RNA-seq data with DESeq2. *Genome Biol.* **15**, 550 (2014).
3. Hale, C. J. *et al.* Identification of Multiple Proteins Coupling Transcriptional Gene Silencing to Genome Stability in Arabidopsis thaliana. *PLoS Genet.* **12**, 1–20 (2016).
4. Potok, M. E. *et al.* Arabidopsis SWR1-associated protein methyl-CpG-binding domain 9 is required for histone H2A.Z deposition. *Nat. Commun.* **10**, 3352 (2019).
