# Supplementary material for: The histone variant H2A.W and linker histone H1 co-regulate heterochromatin accessibility and DNA methylation
Source: Nat Commun. 2021 May 11;12:2683. doi: 10.1038/s41467-021-22993-5 (PMC8113232; doi:10.1038/s41467-021-22993-5)
Supplement: Supplementary file 3 — Reporting Summary [file 41467_2021_22993_MOESM3_ESM.pdf]

nature research

Corresponding author(s): Dr. Olivier Mathieu

Last updated by author(s): Apr 8, 2021

Reporting Summary

Nature Research wishes to improve the reproducibility of the work that we publish. This form provides structure for consistency and transparency in reporting. For further information on Nature Research policies, see Authors & Referees and the Editorial Policy Checklist.

Statistics

For all statistical analyses, confirm that the following items are present in the figure legend, table legend, main text, or Methods section.

n/a	Confirmed	
☐	☒	The exact sample size (n) for each experimental group/condition, given as a discrete number and unit of measurement
☐	☒	A statement on whether measurements were taken from distinct samples or whether the same sample was measured repeatedly
☐	☒	The statistical test(s) used AND whether they are one- or two-sided<br>*Only common tests should be described solely by name; describe more complex techniques in the Methods section.*
☒	☐	A description of all covariates tested
☐	☒	A description of any assumptions or corrections, such as tests of normality and adjustment for multiple comparisons
☐	☒	A full description of the statistical parameters including central tendency (e.g. means) or other basic estimates (e.g. regression coefficient) AND variation (e.g. standard deviation) or associated estimates of uncertainty (e.g. confidence intervals)
☐	☒	For null hypothesis testing, the test statistic (e.g. F, t, r) with confidence intervals, effect sizes, degrees of freedom and P value noted<br>*Give P values as exact values whenever suitable.*
☒	☐	For Bayesian analysis, information on the choice of priors and Markov chain Monte Carlo settings
☒	☐	For hierarchical and complex designs, identification of the appropriate level for tests and full reporting of outcomes
☒	☐	Estimates of effect sizes (e.g. Cohen's d, Pearson's r), indicating how they were calculated

Our web collection on statistics for biologists contains articles on many of the points above.

Software and code

Policy information about availability of computer code

Data collection

Microscope images were collected with ZEN software on a Zeiss Axio Imager Z.1 epifluorescence microscope

Data analysis

The following open source softwares were used:
- R v.3.4.0
- Python 2.7.13
- Trim Galore v.0.5.0
- STAR v.2.7.0a
- htseq-count (HTSeq package v.0.11.2)
- Picard standalone version of Markduplicates (v.1.121)
- DESeq2 v.1.22.2
- bowtie2 v.2.3.4.3 (ChIP-seq and ATAC-seq)
- Genrich v.0.5
- methylpy v.1.4.3
- deeptools v.3.3.0
- BD FACSDiva 8
- ImageJ 1.49v

For manuscripts utilizing custom algorithms or software that are central to the research but not yet described in published literature, software must be made available to editors/reviewers. We strongly encourage code deposition in a community repository (e.g. GitHub). See the Nature Research guidelines for submitting code & software for further information.

Data

Policy information about availability of data

All manuscripts must include a data availability statement. This statement should provide the following information, where applicable:

- Accession codes, unique identifiers, or web links for publicly available datasets
- A list of figures that have associated raw data
- A description of any restrictions on data availability

Data supporting the findings of this work are available within the paper and its Supplementary Information files. A reporting summary for this Article is available as a Supplementary Information file. The datasets and plant materials generated and analyzed during the current study are available from the corresponding author upon request. High throughput sequencing data has been deposited in the Gene Expression Omnibus (GEO) database and can be accessed with the accession number GSE146948 [https://www.ncbi.nlm.nih.gov/geo/query/acc.cgi?acc=GSE146948]. The source data underlying Figures 1b, 1d, 4a, and 5a, as well as Supplementary Figures 1b, 1c, 5a, 6c and 7b are provided as a Source Data file.

Field-specific reporting

Please select the one below that is the best fit for your research. If you are not sure, read the appropriate sections before making your selection.

☒ Life sciences ☐ Behavioural & social sciences ☐ Ecological, evolutionary & environmental sciences

For a reference copy of the document with all sections, see nature.com/documents/nr-reporting-summary-flat.pdf

Life sciences study design

All studies must disclose on these points even when the disclosure is negative.

Sample size	No sample size calculation was performed. Sample sizes were determined based on similar experiments in previous publications. For RNA-Seq, three replicates were used, a standard number in the field. For BS-seq and ChIP-Seq assay, two replicates were used. High degree of overlap/agreement was seen between the replicates. For ATAC-Seq, two replicates were used as these also showed a high degree of overlap.
Data exclusions	No data were excluded from the analysis.
Replication	Two replicates were generated for ChIP-seq and BS-seq and ATAC-seq, and three for RNA-seq. Replicates were highly similar. Other experiments were reproduced in several independent experiments or several biological replicates with technical repeats, as indicated in the figure legends.
Randomization	Seedlings were grown on one or several trays and individuals with the same genetic background were randomly collected to constitute the relevant groups for each replicate.
Blinding	Blinding is not possible since the investigators who analyzed the data also performed the experiments.

Reporting for specific materials, systems and methods

We require information from authors about some types of materials, experimental systems and methods used in many studies. Here, indicate whether each material, system or method listed is relevant to your study. If you are not sure if a list item applies to your research, read the appropriate section before selecting a response.

Materials & experimental systems

n/a	Involved in the study	
☐	☒	Antibodies
☒	☐	Eukaryotic cell lines
☒	☐	Palaeontology
☒	☐	Animals and other organisms
☒	☐	Human research participants
☒	☐	Clinical data

Methods

n/a	Involved in the study	
☐	☒	ChIP-seq
☐	☒	Flow cytometry
☒	☐	MRI-based neuroimaging

Antibodies

Antibodies used	Antibodies used for western: H2A.W.6, H2A, H2A.X, H2A.Z, H2A.W.7, and γH2A.X antibodies were generated in our lab for previously published studies. H2A.W and H1 antibodies were generated in this study. More details can be found in methods section.<br>Antibodies used for ChIP experiments: H2A, H2A.X and H2A.Z antibodies were generated in our lab for previously published studies. H3 (ab1791 Abcam), H3K9me1 (ab8896/Abcam), H3K9me2(ab1220/Abcam), H3K27me1 (17-643/Millipore) and H1 (AS111801/Agrisera) were obtained from commercial sources.

Validation	H2A variants and H1 antibodies were validated by genetic deletion of epitope-encoding gene<br>H2A.W: This study, supplementary figure 1c<br>H2A, H2A.X, H2A.Z and H2A.W.6: Yelagandula et al. (2014) / https://doi.org/10.1016/j.cell.2014.06.006<br>H2A.W.7 and γH2A.X: Lorkovic et al. (2017) / https://doi.org/10.1016/j.cub.2017.03.002<br><br>H1 used for ChIP: The manufacturer states it reacts with Arabidopsis thaliana. First validation:She et al. (2013) / https://doi:10.1242/dev.095034 . Validation for ChIP: Wollmann et al. (2017) / https://doi.org/10.1186/s13059-017-1221-3<br>H1 used for western blot: https://doi.org/10.1038/s41556-021-00658-1<br>H3(ab1791/Abcam): The manufacturer states it is applicable to western blot and ChIP in many species including Arabidopsis thaliana<br>H3K9me1(ab8896/Abcam): The manufacturer states it is ChIP-grade. Validation for Arabidopsis: https://doi.org/10.1111/j.1365-313X.2010.04400.x<br>H3K9me2(ab1220/Abcam): The manufacturer states it is a ChIP-grade antibody and works with many species including Arabidopsis thaliana<br>H3K27me1(17-643/Millipore): The manufacturer states it is ChIP-grade. Validation for Arabidopsis:https://doi.org/10.1038/s41556-020-0515-y

ChIP-seq

Data deposition

☒ Confirm that both raw and final processed data have been deposited in a public database such as GEO.

☒ Confirm that you have deposited or provided access to graph files (e.g. BED files) for the called peaks.

Data access links<br>*May remain private before publication.*	Raw and processed data were deposited in GEO with accession number GSE146948.
Files in database submission	For all datasets: raw reads in fastq format. For RNA-seq, we additionally provide the output of DESeq2 analysis, including adjusted p-values, estimated log2fc, etc. For ATAC-seq and ChIP-seq, we also provide coverage tracks as bigWig files. For BS-seq, we provided per-position methylation data.
Genome browser session<br>(e.g. UCSC)	no longer applicable

Methodology

Replicates	RNA-seq experiments were performed in triplicate, ATAC-seq in duplicate and ChIP-seq and BS-seq in duplicate. There was good agreement between all replicates.
Sequencing depth	Reads obtained and mapping statistics are available in Supplementary Table 3. RNA-seq and ATAC-seq data were 75bp PE, ChIP-seq were 75 bp SE, and BS-seq were either 100 bp or 150 bp PE.
Antibodies	Antibodies used for ChIP experiments: H2A, H2A.X and H2A.Z antibodies were generated in our lab for previously published studies. H3 (ab1791 Abcam), H3K9me1 (ab8896/Abcam), H3K9me2(ab1220/Abcam), H3K27me1 (17-643/Millipore) and H1 (AS111801/Agrisera) were obtained from commercial sources.
Peak calling parameters	Peak calling was not used in our analysis.
Data quality	We observed good library mapping rates and agreement between replicates.
Software	ChIP-seq data were aligned using bowtie2 v.2.3.4.3, and coverage tracks were generated using deeptools v.3.3.0.

Flow Cytometry

Plots

Confirm that:

☒ The axis labels state the marker and fluorochrome used (e.g. CD4-FITC).

☒ The axis scales are clearly visible. Include numbers along axes only for bottom left plot of group (a 'group' is an analysis of identical markers).

☒ All plots are contour plots with outliers or pseudocolor plots.

☐ A numerical value for number of cells or percentage (with statistics) is provided.

Methodology

Sample preparation	Briefly, 0.5 g of freshly collected 10-day old seedlings were chopped in 4 ml of pre-chilled lysis buffer (15 mM Tris-HCl pH 7.5, 20 mM NaCl, 80 mM KCl, 0.5 mM spermine, 5mM ß-mercaptoethanol, 0.2% Triton X-100). After chopping, the suspension was filtered through a 40 µM filter. Nuclei were further enriched using a sucrose gradient. Enriched nuclei were resuspended in 0.5

	ml of pre-cooled lysis buffer with 4,6-Diamidino-2-Phenylindole (DAPI) and incubated for 15 min. DAPI stained nuclei were FACS analyzed.
Instrument	FACS Aria III (BD Biosciences)
Software	BD FACSDiva 8. For generating illustration in supplementary fig 4, FCS Express 7 (free demo version)/De Nova software is used.
Cell population abundance	Nuclei isolated from Arabidopsis seedlings. Nuclei suspension also contains chloroplasts.
Gating strategy	Nuclei were gated for shape (Area FSC-Area SSC), doublets (Area FSC-Height FSC). Based on DAPI signal intensity nuclei content was determined.

☒ Tick this box to confirm that a figure exemplifying the gating strategy is provided in the Supplementary Information.
